# Extreme MHC class I diversity in the sedge warbler (*Acrocephalus schoenobaenus*); selection patterns and allelic divergence suggest that different genes have different functions

**DOI:** 10.1186/s12862-017-0997-9

**Published:** 2017-07-05

**Authors:** Aleksandra Biedrzycka, Emily O’Connor, Alvaro Sebastian, Magdalena Migalska, Jacek Radwan, Tadeusz Zając, Wojciech Bielański, Wojciech Solarz, Adam Ćmiel, Helena Westerdahl

**Affiliations:** 1 Institute of Nature Conservation, Polish Academy of Sciences, Al. Mickiewicza 33, 31-120 Kraków, Poland; 2 Molecular Ecology and Evolution Lab, Department of Biology, Lund University, Ecology Building, Sölvegatan 37, 223 62 Lund, Sweden; 3 Evolutionary Biology Group, Faculty of Biology, Adam Mickiewicz University in Poznań, ul. Umultowska 89, 61-614 Poznań, Poland

**Keywords:** MHC class I, Sedge warbler, Extreme diversity, Different functions of loci

## Abstract

**Background:**

Recent work suggests that gene duplications may play an important role in the evolution of immunity genes. Passerine birds, and in particular Sylvioidea warblers, have highly duplicated major histocompatibility complex (MHC) genes, which are key in immunity, compared to other vertebrates. However, reasons for this high MHC gene copy number are yet unclear. High-throughput sequencing (HTS) allows MHC genotyping even in individuals with extremely duplicated genes. This HTS data can reveal evidence of selection, which may help to unravel the putative functions of different gene copies, i.e. neofunctionalization. We performed exhaustive genotyping of MHC class I in a Sylvioidea warbler, the sedge warbler, *Acrocephalus schoenobaenus*, using the Illumina MiSeq technique on individuals from a wild study population.

**Results:**

The MHC diversity in 863 genotyped individuals by far exceeds that of any other bird species described to date. A single individual could carry up to 65 different alleles, a large proportion of which are expressed (transcribed). The MHC alleles were of three different lengths differing in evidence of selection, diversity and divergence within our study population. Alleles without any deletions and alleles containing a 6 bp deletion showed characteristics of classical MHC genes, with evidence of multiple sites subject to positive selection and high sequence divergence. In contrast, alleles containing a 3 bp deletion had no sites subject to positive selection and had low divergence.

**Conclusions:**

Our results suggest that sedge warbler MHC alleles that either have no deletion, or contain a 6 bp deletion, encode classical antigen presenting MHC molecules. In contrast, MHC alleles containing a 3 bp deletion may encode molecules with a different function. This study demonstrates that highly duplicated MHC genes can be characterised with HTS and that selection patterns can be useful for revealing neofunctionalization. Importantly, our results highlight the need to consider the putative function of different MHC genes in future studies of MHC in relation to disease resistance and fitness.

**Electronic supplementary material:**

The online version of this article (doi:10.1186/s12862-017-0997-9) contains supplementary material, which is available to authorized users.

## Background

Gene duplication plays an important role in evolution [1]. New gene copies, known as paralogs, create redundancy which enables them to evolve new functions as the evolutionary constraint of maintaining the original gene function is released [2]. This concept of duplicated gene copies acquiring new functions, referred to as neofunctionalization, has long been established, and there are several recent examples [1–4]. Regions of highly duplicated genes, such as the Major Histocompatibility Complex (MHC) genes, have been suggested to evolve by several different processes one being the so called ‘birth and death process’ where some gene copies are very old, others gain slightly new functions and finally some become non-functional through mutations and deletions [2, 5]. Current molecular genetic techniques make it possible to accurately genotype highly duplicated genes [6–10], like the MHC genes, and it is now evident that the number of MHC gene copies varies considerably between different taxa [11].

Classical MHC genes encode molecules of central importance in vertebrate adaptive immunity [12]. These MHC molecules are responsible for presenting antigens (peptides) to the immune system for recognition and elimination [13]. There are two main classes of classical MHC genes involved in adaptive immunity, class I and class II. Class I genes encode MHC class I molecules that present peptides (antigens e.g. from viruses) from the intracellular environment to t-cells, whereas class II genes encode molecules that present peptides (e.g. antigens from many bacteria) from the extracellular environment to t-cells [12, 13]. Classical MHC genes that encode antigen presenting MHC molecules are some of the most polymorphic genes currently characterised [14]. The high polymorphism in these classical genes is believed to be maintained by balancing selection driven by pathogens and mate choice [15–17]. The role of pathogen pressure in shaping MHC diversity has been empirically demonstrated by a number of studies [18–22] and there is also empirical support for the role of mate choice in maintaining MHC diversity [23–25]. However, there is also a group of MHC genes that have limited expression, low polymorphism and a less clear role in immunity, and these genes are referred to as non-classical MHC genes [26].

The number of MHC genes per individual differs greatly both between and even within species [11, 16]. In birds, chickens *Gallus gallus* of the order Galliformes, have a “minimal essential MHC”, i.e. a MHC region with smaller introns, denser gene regions and considerably lower diversity than human MHC [27, 28]. A similar MHC structure has been reported in other galliform birds, which have between one and three classical MHC class I gene copies [29–31]. Birds of the order Passeriformes are known to have more complex MHC systems, both for class I and class II genes, with many extremely polymorphic and highly duplicated genes, long introns and pseudogenes [8, 20, 24, 25, 32, 33].

Genetic footprints, i.e. patterns of past selection, that differ between groups of MHC genes, such as classical and non-classical MHC genes, indicate that these groups of genes have evolved divergent functions. In addition, MHC class I alleles of several different lengths, due to indels within exon 3, have been demonstrated in a number of bird species [25–29].

There is, for example, evidence from species within the genus *Passer* that MHC class I alleles with a 6 bp deletion in exon 3 are putatively non-classical, since they have low polymorphism and very limited evidence of positive selection [34]. In contrast, in domestic chicken classical MHC class I genes have a 3 bp deletion whereas non-classical class I genes contain no deletions in exon 3 [35]. So far, there is no consistent pattern relating the presence or absence of deletions in exon 3 to the occurrence of classical and non-classical genes in birds. Further studies are needed to confirm/reject the importance of such deletions in relation to gene function. Still, selection pressures are likely to generate genetic footprints that separate classical and non-classical MHC genes and warrants further investigation in additional bird species.

The aim of this study was to use evidence of past selection in combination with present allelic distributions to better understand the putative functions of the extremely duplicated MHC genes in passerine birds and potentially unravel the process of neofunctionalization. A recent study by O’Connor et al. [36] showed that there is a strong phylogenetic signal in MHC diversity (number of MHC alleles per individual) in the parvorder Passerida and that species within the superfamily Sylvioidea have particularly high MHC diversity. Biedrzycka et al. [10] recently optimized HTS genotyping for MHC class I in sedge warblers *Acrocephelus shoenobaenus*, a Sylvioidea species, and we therefore decided to further characterize the MHC diversity in this species.

We partly characterized MHC class I genes in sedge warblers by Sanger sequencing transcripts (complementary DNA (cDNA)). We then used three different sets of primers on both genomic DNA (gDNA) and cDNA to capture the full polymorphism in these highly duplicated genes using HTS. With this information in hand we designed specific PCR primers for HTS (MiSeq Illumina) that would amplify the majority of all alleles (sequence variants), while excluding known pseudogenes. We genotyped exon 3 of MHC class I alleles (the antigen binding site of the MHC class I molecule is encoded by exons 2 and 3) in 863 individuals from a study population in the Nida wetlands, Poland. We found that sedge warblers have the highest MHC class I diversity of any passerine bird studied to date and demonstrate distinct differences between the signatures of selection on alleles with and without deletions. These genetic footprints imply divergent functions for alleles with and without deletions and thus partly unravel why passerine species, such as the sedge warbler, have so many MHC class I genes.

## Methods

### Sample collection and DNA and RNA extraction

Samples were collected in a sedge warbler population from the Nida wetlands (a natural wetland situated in central Poland, 20°28′–20°32′ E, 50°33′–50°35′ N) during the 2004–2012 breeding seasons. The ecology of this population has been studied for 20 consecutive years and every year it is subject to systematic monitoring with all breeding birds marked individually [37]. Field procedures were carried out under the permits from the Local Ethical Committee in Krakow (nos.: 37/OP/2004 and 24/2010). Sedge warblers were mist-netted and blood samples were obtained from the brachial veins of 915 adult individuals of both sexes. The blood samples were preserved in 95% ethanol. Additionally, blood samples were collected from four unrelated sedge warblers (individuals A, B, C and D). These blood samples were placed in RNAprotect Animal Blood Tubes (QIAGEN) and stored at 4 °C overnight prior to RNA extraction. Genomic DNA was extracted with the Nucleospin Tissue Kit (Macherey-Nagel, Germany). Total RNA was isolated from blood with RNAzol (QIAGEN) and cDNA was obtained by reverse transcription that was carried out with Omniscript Reverse Transcription Kit (QIAGEN).

### Characterization of MHC class I in sedge warblers

#### Long reads using Sanger sequencing on cDNA transcripts

To obtain long sequences (exons 2–4) of expressed sedge warbler MHC class I alleles, cloning and Sanger sequencing was performed twice on cDNA from each of two individuals (individuals A and B). This approach enabled us to get high quality DNA sequence information and an initial estimate of genetic variation in MHC class I genes in sedge warblers (individuals A and B were also used in the later described 454-procedure). The cDNA was amplified using the degenerate primers PadoM2 and PadoM4 which were designed to amplify the region spanning exons 2, 3 and 4 of MHC class I in house sparrows ([34]). Amplification was performed with HotStar Master Mix (QIAGEN) and the reactions were run for 30 cycles at 95 °C for 30 s, 66 °C for 30 s, 72 °C for 90 s. PCR products were purified with MinElute PCR Purification Kit (QIAGEN), ligated in a plasmid vector and transformed into bacteria using StrataClone PCR Cloning Kit (Stratagene). Thirty-six clones from each cloned product (individuals A and B each subject to two independent PCR amplifications, i.e. 144 clones in total) were randomly selected for bidirectional sequencing by dye terminator cycle sequencing (BigDye 3.1, Life Technologies) and resolved on ABI PRISM 3130xl automated sequencer (Applied Biosystems). The sequences were aligned and edited in BioEdit Sequence Editor [38]. Only alleles found in both PCRs from the same individual were treated as true alleles.

#### Long reads using the 454 amplicon sequencing

To obtain additional knowledge about MHC variation in sedge warblers, prior to designing specific exon 3 primers (see Illumina MiSeq), we used three different pairs of primer pairs that amplify: parts of exons 2 and 3 (primer combination 1, HN30-HN40), exon 3 (primer combination 2, HNalla-HN46) and parts of exons 3 and 4 (primer combination 3, HN11-HN22) (Additional file 1: Figure S1, Additional file 2: Table S1; Westerdahl et al. 2004). Each primer contained a 6 bp tag that indicated sample identity. These amplifications were performed on both cDNA and gDNA from individuals A, B, C and D. The amplification conditions were as described in Westerdahl et al. [33] with an annealing temperature of 65 °C for all amplicons. The concentration of the PCR products were estimated through the strengths of the bands in agarose gel electrophoresis, and PCR products of the same length were pooled into approximately equimolar quantities. The resulting pools were purified using the MinElute PCR Purification Kit (QIAGEN). Purified pools were then sequenced as a part of a single 454 FLX run according to the 454 Amplicon Sequencing protocols provided by the manufacturer (Roche 454) at the Functional Genomics Center, Uni ⁄ETH Zurich. The sequencing output was analysed using jMHC software [39]. The jMHC software extracts reads according to primer sequence and tags that indicate individual identity and generates a table of variants (alleles) present in each individual along with the number of reads representing each variant. Variants lacking complete primer or tag sequences as well as those with ambiguous base pairs were removed using jMHC prior to further analysis. The gDNA and cDNA fragments were aligned and variants present in less than 10 copies per amplicon were removed. Variants that appeared to be chimeras of two more abundant variants present in the amplicon and variants that differed by 1-2 bp from more abundant variants were also removed. This simplified approach to remove artefacts was employed as retained variants were primarily used for primer design, and not for genotyping.

#### Designing exon 3 primers for genotyping with Illumina MiSeq

Our aim was to design a primer pair that would amplify MHC class I exon 3 in all alleles in open reading frame in sedge warblers. We choose to amplify exon 3 as it holds part of the peptide binding region (PBR) and has the highest diversity in MHC class I genes. With the knowledge obtained from the Sanger and 454 sequencing (above) we designed a pair of degenerate primers (HNallaN-Fwd: GAGYGGGGGTCTCCACAC and HN46N-Rev.: TGCGMTCCAGYTCCTTCTGCCC) to amplify exon 3 (Additional file 1: Figure S1, 235-241 bp). We used the information on polymorphisms in the sequences flanking exon 3 to avoid amplification of a set of non-functional alleles with an 11 bp deletion that were previously identified with 454 sequencing. First we performed 454 sequencing on amplicons obtained with the primers HNallaN-Fwd and HN46N-Rev. on gDNA and cDNA from the four sedge warblers mentioned above (individual A, B, C and D) and then we performed Illumina MiSeq (see next paragraph).

### Genotyping of MHC class I in sedge warblers

#### Illumina sequencing, MiSeq

Illumina sequencing was performed on amplicons obtained with the primers HNallaN-Fwd and HN46N-Rev. on genomic DNA samples from 915 sedge warblers. Illumina sequencing primers contained a unique 6 bp barcode in addition to a template specific primer. The amplifications were performed in 96-well PCR plates with 6 negative controls per PCR plate. Each plate was amplified with a combination of 12 forward and eight reverse, uniquely barcoded, primers such that each sample was amplified with a unique tag combination, similar to Galan et al. (2010). The amplification procedure was performed as in Biedrzycka et al. [10] The PCR amplicons from two 96-plates were pooled in one sequencing run to get a coverage of 20,000–100,000 reads per *amplicon/*individual (the sedge warbler samples constituted around 80% of the run). Genotyping was performed with AmpliSAS software [40] exactly as described in [10]. A maximum of 20,000 reads per amplicon were used for genotyping to reduce computational load.

### Tests of allelic distances, historical selection, recombination and networks

The unique MiSeq sequences were aligned manually first using BioEdit and then further edited and interpreted using Geneious 7.1 [38, 41]. P-distances of nucleotides and amino acid sequences were estimated using MEGA7 [42]. To describe phylogenetic relationships among MHC class I alleles we used phylogenetic networks. When alleles are created by processes involving gene duplication and loss, recombination and gene conversion, phylogenetic networks are a better alternative than phylogenetic trees that present only one possible topology as representations of relationships among alleles [43]. We constructed a network of alleles found in individuals A, B, C and D for which cDNA and gDNA was amplified. The network was intended to reveal any evidence of clustering between alleles based upon length and/or transcription as well as general divergence between alleles. The neighbor-net was constructed in SplitsTree v.4 [44] software with edge weights estimated using ordinary least squares variance and a threshold of 10^−6^. Maximum likelihood model selection of nucleotide substitutions was performed in MEGA 7 [42] and used for network construction. The best selected model was K2P + G(0.29). Bootstrap support with 1000 replicates was provided, but presented only for the most significant splits, with the bootstrap support higher than 70.

We tested selection acting on MHC class I exon 3 in sedge warblers using several different approaches and these selection tests were performed separately on alleles of different lengths (no deletion, 3 bp deletion and 6 bp deletion alleles). As recombination events can affect the outcome of selection tests, we first tested for evidence of recombination. Recombination tests were performed in RPD v4.80 [45] using RDP, GENECONV, Chimaera and MaxChi tests. A recombination event was considered to have occurred if we obtained significant recombination results for at least three of the tests performed. Positive selection was estimated using FEL, REL, FUBAR and MEME tests in the HyPhy 2.2.4. software [46]. As the results of these tests could differ from one another, we only considered a site to be under positive selection if this was indicated by at least three out of four tests. To do this we first ran a model selection procedure to identify the model that best fit the data. Neighbour-Joining trees using the maximum likelihood method were then used to obtain branch lengths and substitution rates [47].

As the number of alleles of different lengths was considerably different (3133 alleles with no deletion, 386 alleles with a 6 bp deletion and 47 alleles with a 3 bp deletion) we re-ran selection tests on sub-sampled dataset, to check whether the outcomes of the selection tests were influenced by sample size. We performed 100 randomized tests by sub-sampling 47 alleles containing a 6 bp deletion and 47 alleles with no deletion. Forty-seven alleles were selected as this matched the total number of alleles detected that contained a 3 bp deletion. We used the MEME test to estimate the number of positively selected sites on each of these 100 subsets of 47 alleles. We then compared the distribution of the number of positively selected sites detected with the results of the MEME tests performed in the full dataset for each allele length. Only the MEME test of selection was used for these analyses. MEME applies mixed effects model of evolution and is known to perform well for detecting both episodic and pervasive selection at the level of individual site. It is recommended for large alignments in which purifying selection acting on specific sites in some lineages can mask an effect of positive selection in other lineages [48].

We genotyped MHC exon 3 alleles in individuals that were present in our study population between 2004 and 2011 and visualized the frequency distributions of alleles with no deletions, those with a 3 bp deletion and alleles with a 6 bp deletion graphically in Microsoft Excel combining the alleles from all years.

## Results

### Long cDNA reads from Sanger sequencing

We obtained 12 verified 729-735 bp MHC class I transcripts from two sedge warbler individuals (partial exon 2 to exon 4, i.e. the α1 to α3 domains 243–245 amino acids (aa), ass.no. KM-014676-KM014687, (Additional file 3: Figure S2)). Two of the 12 cDNA sequences had a 6 bp nucleotide deletion at amino acid position 147–148 in the α2 region (exon 3, Additional file 3: Figure S2). The sedge warbler cDNAs were easily aligned to previously published MHC class I transcripts from other songbirds and many conserved sites were verified (e.g. cysteines (C) at positions 99, 163 and 201, sites known to bind the C- and N-terminal of peptides at positions 58Y, 141 T, 145 W, 158Y and 170Y, and the CD8 binding sites at positions 220–244). Interestingly, two supposedly conserved sites were variable, 83R/83 W and 144R/144 L.

### Genotyping of cDNA and gDNA using 454 amplicon sequencing

MHC class I exon 3 from four individuals was amplified from both cDNA and gDNA. In total 96 alleles in open reading frame were verified: 58 alleles were transcribed, i.e. were present in both cDNA and gDNA, whereas 38 alleles were putatively non-expressed since they were only present in gDNA. Three of these 38 non-expressed alleles contained stop codons. The four genotyped individuals had 26–36 gDNA alleles and 18–25 cDNA alleles (individual 1: 26 gDNA alleles and 18 cDNA alleles, individual 2: 36 and 25, individual 3: 27 and 18 and individual 4: 34 and 19). On average 65% of the alleles in open reading frame were transcribed.

The exon 3 alleles were of three different lengths; alleles containing no deletion (241 bp, 80 amino acids (aa)), alleles with a 3 bp deletion (238 bp, 79aa) and alleles with a 6 bp deletion (235 bp, 78aa). Eighty alleles had no deletion, five alleles had a 3 bp deletion and 11 alleles had a 6 bp deletion The percentage of transcribed alleles was higher for alleles with a deletion (3 bp deletion 80% and 6 bp deletion 73%) than for the alleles with no deletion (60%). These four individuals were also re-sequenced from gDNA using Illumina MiSeq, and there was high repeatability between methods (454 and MiSeq) for individual 1 (96%), individual 2 (100%) and individual 3 (100%), while the repeatability was lower for individual 4 (78%). The discrepancy in repeatability is to a large extent explained by the lower coverages in 454 amplicons than MiSeq amplicons (see below).

### Genotyping of MHC class I exon 3 (gDNA) using MiSeq

MHC class I exon 3 was genotyped successfully in 863 out of 915 blood-sampled individuals using MiSeq. Altogether, 3566 sequences were verified as putative exon 3 alleles in open reading frame with 2018 of these alleles being found in at least two individuals (GenBank acc. nr.: KP706831 - KP707785, KP707787–708145, KP708147 - KP708431, KP708433 - KP708535, KP708537 - KP708536 and KU702953 – KU704816).

### Diversity and phylogenetic relationships of MHC class I exon 3 alleles

**Fig. 1:**
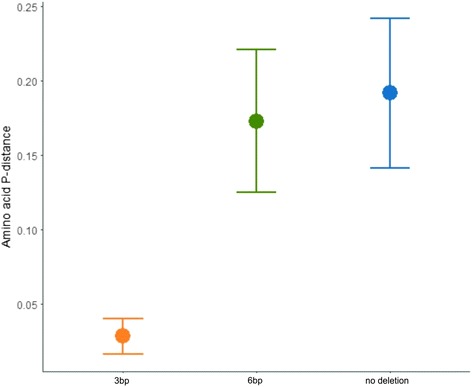
Average allelic divergence (amino acid p-distance, mean ± s.d) of sedge warbler MHC class I exon 3 alleles of three different lengths; 3 bp deletion alleles (*orange*), 6 bp deletion alleles (*green*) and alleles with no deletion (*blue*)

The 3566 nucleotide sequences translated to 2760 unique amino acid sequences. As with the aforementioned 454 run, the alleles were of three different lengths (no deletion, 3 bp deletion and 6 bp deletion, Table 1). Alleles containing no deletion and alleles with a 6 bp deletion were more polymorphic (π = 0.126 for alleles with no deletion and π = 0.108 for alleles with a 6 bp deletion) than alleles with a 3 bp deletion (π = 0.016), and the divergence, i.e. nucleotide and amino acid p-distances, were a magnitude higher for these alleles than for alleles with a 3 bp deletion (Table 1; Fig. 1).

**Table 1:** Diversity and divergence estimates of sedge warbler MHC class I exon 3 alleles, alleles with no deletion, 3 bp deletion alleles and 6 bp deletion alleles, the estimates are reported individually as well as combined for all sequence lengths

Exon 3 sequences	Number of nucleotide sequences	Nucleotide p-distance	Number of positively selected sites	Number of amino acid sequences	Amino acid seq. Per nucleotide seq. Ratio
No deletion alleles	3133	0.128	18	2413	0.77
3 bp deletion	47	0.016	0	32	0.68
6 bp deletion	386	0.111	8	314	0.81
All exon 3 sequences	3566	0.130	-	2760	0.77

**Fig. 2:**
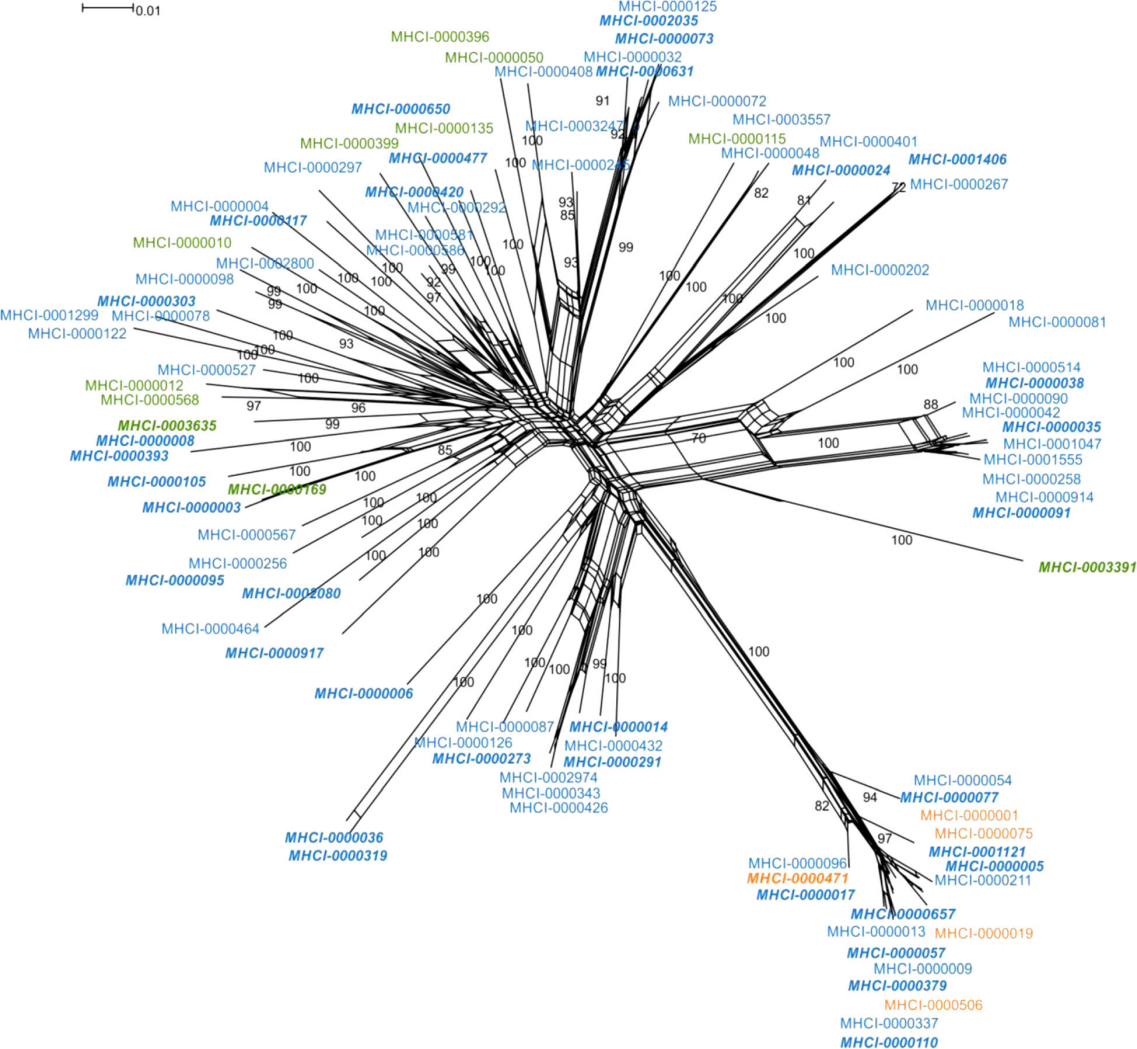
Neighbor-net network of sedge warbler MHC class I exon 3 alleles constructed from all cDNA and gDNA alleles detected in four individuals, alleles marked with bold and italics were found only in gDNA amplicons. Bootstrap support values (based on 1000 replicates) higher than 70% are presented. The loops imply areas of phylogenetic uncertainty or reticulations. 3 bp deletion alleles are shown in *orange*, 6 bp deletion alleles in *green* and alleles with no deletion in *blue*

The total number of alleles per individual ranged from 12 to 65 with a mean of 36.3 (SD = 8.2) suggesting the presence of at least 33 MHC class I genes in some individuals, assuming heterozygosity. All individuals had alleles with no deletion (mean 31.4 (7.5), range 8–59), 93% of the individuals carried alleles with a 6 bp deletion (mean 3.6 (1.9), range 1–12) and 99% of the individuals carried alleles with a 3 bp deletion (mean 1.6 (0.86), range 1–7). A phylogenetic network of all alleles that were genotyped in cDNA and/or gDNA amplicons from four individuals did not reveal any clustering separating expressed (cDNA) and non-transcribed alleles (gDNA), neither, was there any strict clustering based on allelic lengths (Fig. 2). However, the five alleles with a 3 bp deletion were all found in a single significantly supported cluster (bootstrap support of 100), though there were also several alleles with no deletion in this cluster.

### Tests of selection

Prior to testing for selection we tested for recombination in order to know if signs of positive selection could be confounded by recombination [49]. We ran the tests of recombination using the 50 most frequent alleles (consisting of 44 alleles with no deletion, four 6 bp alleles and two 3 bp alleles) and we found one recombination event between no deletion and 6 bp deletion alleles, but the event was significant only in two out of four tests. Then we did recombination tests separately for the three allelic lengths. We detected no recombination among the 3 bp deletion alleles, and found only a single recombination event among 6 bp alleles and likewise a single recombination event among the alleles with no deletion. However, these single recombination events were only significant in two out of four tests, and hence the results show that overall recombination is unlikely to have any large effects on the tests for positively selected sites.

**Fig. 3:**
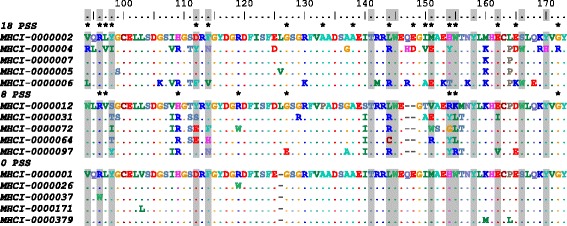
Amino acid alignment of sedge warbler MHC class I exon 3 alleles of three different lengths, alleles with no deletion, 6 bp deletion alleles and 3 bp deletion alleles. The five most frequent alleles of each allelic length are presented. Deletions are indicated with minus signs (−), the peptide binding region is indicated by *grey* boxes (inferred from HLA-A, Björkman et al. 1987) and positively selected sites with an asterisk (*). Alleles with no deletion had 18 positively selected sites (PSS), alleles with a 6 bp deletion had eight PSS and alleles with a 3 bp deletion had no PSS

The analyses on selection showed that alleles with a 3 bp deletion had no positively selected sites, the alleles without any deletions had 18 positively selected sites (10 of these were within the PBR) and alleles with a 6 bp deletion had eight positively selected sites (only three were in the PBR). Six of these eight positively selected sites in alleles with a 6 bp deletion were shared with the alleles containing no deletion (Fig. 3, Table 1). In order to investigate if the number of alleles tested could explain the number of sites subject to positive selection we re-ran selection tests on 100 sets of 47 randomly selected alleles that contained no deletion and then repeated this procedure for alleles containing a 6 bp deletion. There were between eight and 16 positively selected sites among the no deletion alleles and between five and 14 among the 6 bp deletion alleles, hence although the number of selected sites varied a little depending on sample size it is still clear that these alleles have many more sites subject to positive selection than the 3 bp deletion alleles (Additional file 4: Figure S3).

**Fig. 4:**
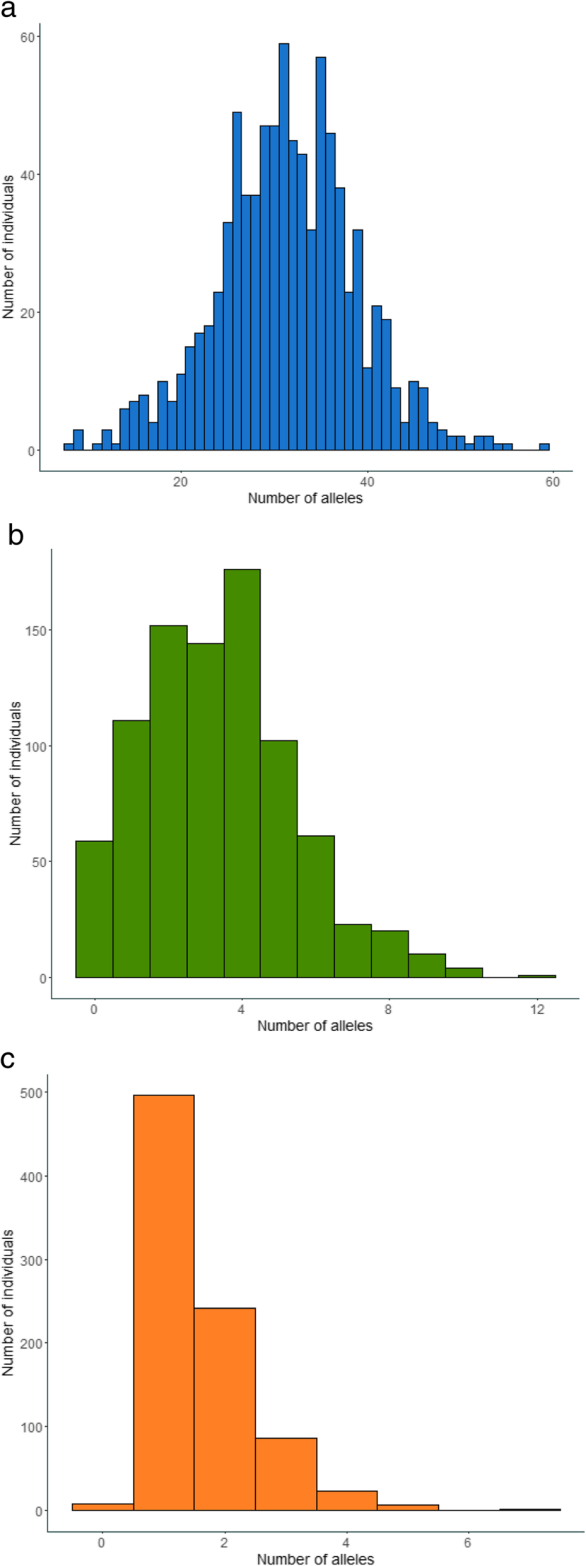
Frequency distributions of MHC class I exon 3 alleles in our study population of sedge warblers, presented separately for **a**) alleles with no deletion (*blue*), **b** 6 bp deletion alleles (*green*) and **c**) 3 bp deletion alleles (*orange*)

There was a skewed distribution in the number of alleles possessed by individuals for the 3 bp deletion alleles compared to the alleles with no deletion and the 6 bp deletion alleles in our study population (Fig. 4). There were only 47 different 3 bp deletion alleles but more or less every individual had one or two 3 bp deletion alleles (99% of the individuals had 3 bp deletion alleles). All analyses suggested that the 3 bp deletion alleles have characteristics different from the alleles with no deletion and with a 6 bp deletion: they were in a significantly supported cluster in the network, had low diversity and divergence, no positively selected sites and a skewed distribution in our study population (Table 1, Figs. 1, 2, 3 and 4).

## Discussion

Here we describe, to the best of our knowledge, the highest number of MHC class I alleles ever reported in any passerine species, a group which is notable for its extraordinary MHC diversity [7, 8, 10, 24, 33, 34, 50, 51]. In addition, we present evidence for striking variation in the signatures of selection between groups of alleles based upon their lengths, i.e. alleles with either no deletions, a 3 bp deletion or a 6 bp deletion, raising the possibility that some groups of MHC class I alleles have evolved functions other than that of classical antigen presentation. According to our findings, based on exon 3 sequences, a single sedge warbler individual can carry between 12 and 65 MHC class I alleles, on average 36 alleles. This demonstrates that MHC class I genes are highly duplicated in sedge warblers and that the number of alleles varies greatly between individuals. As we made a considerable effort to thoroughly characterize the variation in regions flanking MHC class I exon 3 in the sedge warbler, we find it unlikely that the primer design would create such a bias in allelic number between individuals. Hence, this variance in the number of MHC class I alleles per individual should, to a large extent, be due to gene copy number variation between individuals, which has been previously demonstrated in other bird species [8, 52].

Despite many papers describing extraordinary levels of MHC diversity in passerines [7, 8, 24, 33, 51], explanations for this phenomenon are scarce. It is likely that MHC genes evolve through a birth-and-death process [2]. The diversity and size of the MHC gene family would then depend on the trade-off between stochastic birth-and-death process and the need to maintain genes that carry out necessary immune functions [2, 53]. Alcaide et al. (2013) [54] provided evidence that MHC class I genes are under stronger adaptive selection in passerines compared to non-passerine birds. They proposed that this may reflect a ‘general syndrome of songbird evolution’ characterized by having a small body size, rapid rate of evolution due to their short generation time and occupying a diverse range of habitats. Recent work from Sackton et al. (2017) [55] in insects suggests that pathogen diversity may drive the duplication of immunity genes, i.e. species that are exposed to selection from a wider range of pathogens may have a higher number of immune gene copies. If we put the ideas from Alcaide et al. (2013) and Sackton et al. (2017) into the context of the highly diverse MHC genes in passerines, it could be speculated that high MHC diversity is associated with passerines successfully occupying a wide range of habitats. Rapid rates of adaptive evolution may have facilitated the accumulation of high MHC diversity. Similarly, high MHC diversity is likely to be maintained if it facilitates the occupation of novel habitats with novel pathogens, given that a large number of MHC gene copies are advantageous in most host-pathogen interactions.

The high number of MHC class I alleles per individual in sedge warblers raises the question of whether all the alleles encode antigen presenting MHC molecules. All alleles in open reading frame in the 454 and MiSeq datasets had classical sites of MHC class I genes, i.e. those involved in formation of disulphide bridges. In addition, more than 60% of these alleles were expressed (transcribed). Thus, it seems that sedge warblers have a very high number of putatively expressed MHC class I alleles. The data in this study only allowed us to test whether or not alleles were expressed and not the degree to which they were expressed. Measuring the degree of gene expression could have been informative since non-classical MHC genes often have lower expression than classical MHC genes. Hence, measuring gene expression could have distinguished classical and non-classical genes. Large differences in the degree to which different MHC genes are expressed have been found in birds from several different orders e.g. in Galliformes and Anseriformes [28, 29, 56]. A recent study suggests that such differences in expression of different MHC class I genes exist also in birds from the order Passeriformes (Drews et al. Submitted). We therefore find it likely that there are differences in MHC class I gene expression in sedge warblers, though this has not yet been tested.

We found three different lengths of MHC class I alleles in sedge warblers, alleles with no deletion (241 bp) and those containing either a 3 bp (238 bp) or a 6 bp deletion (235 bp). Two other closely related *Acrocephalus* warblers, the reed warbler *Acrocephalus scirpaceus* and the moustached warbler *A. melanopogon* also have MHC class I alleles with this 6 bp deletion ([36], O’Connor et al. unpublished data), while a third, more distantly related, Acrocephalus warbler, the great reed warbler *A. arundinaceus*, does not [33]. A similar 6 bp deletion, putatively in the same position, has been reported in MHC class I alleles in three sparrow species within the genus *Passer* [34, 57]. In contrast to sedge warblers, the alleles with a 6 bp deletion in sparrows are likely to be non-classical [34, 57]. Therefore, in addition to possessing a high number of MHC genes, passerine species appear to display intriguing variation in the characteristics, both considering indels and polymorphisms, of their MHC genes. The 6 bp deletion alleles have so far only been reported in the genera *Passer* and *Acrocephalus* within Passerida [34, 36] and these bird groups diverged around 45 MYA [58]. As the alleles containing a 6 bp deletion have such different characteristics, i.e. putatively classical genes in *Acrocephalus* and non-classical in *Passer*, we believe that these deletions probably have independent evolutionary origins. The sedge warbler MHC alleles with a 6 bp deletion did not form any significant separate clusters in the network analyses, which is again in contrast to findings of Karlsson and Westerdahl 2016 [34] where *Passer* 6 bp deletion alleles clustered together.

We only detected weak signals of recombination between MHC alleles in the sedge warbler. However, recombination tests may have limited power to detect recombination events when recombination is frequent and the recombination blocks are narrow, which seems to be the case for MHC genes [59]. Such micro-recombination events could be one explanation for why alleles of different lengths are found within the same cluster in the network analyses. An alternative explanation is that alleles containing a 3 bp or 6 bp deletion have arisen several times in different gene copies. As alleles cannot be assigned to particular loci from our data, it is not possible to discriminate between these two potential explanations for why alleles of different lengths cluster together. However, given that micro-recombination is common in MHC genes [58], we find the former explanation the most probable.

Unlike the alleles with a 6 bp deletion, the alleles with a 3 bp deletion in sedge warblers showed distinct characteristics suggesting that they are not classical MHC genes. The 3 bp deletion alleles had low diversity and low divergence compared to the alleles with no deletion and 6 bp deletion alleles. Moreover, the alleles with a 3 bp deletion showed no evidence of any positively selected sites. In contrast, the alleles without deletion and 6 bp deletion alleles had 18 and 8 positively selected sites, respectively. These findings suggest that the 3 bp deletion alleles are subject to different selection pressures than the alleles with no deletion and 6 bp deletion alleles. The 3 bp deletion alleles had a skewed distribution in the population data set and the vast majority of the individuals (99%) carried at least one such allele. The fact that so many individuals carry one or more 3 bp deletion alleles implies that there is selection favoring the possession of at least one of these alleles. It should be noted, that with our genotyping protocol we can only register the presence or absence of specific alleles, hence we cannot separate individuals that are homozygotes and heterozygotes, respectively for 3 bp deletion alleles.

The strikingly low diversity of the 3 bp deletion alleles suggests that they may represent non-classical MHC genes. Five different alleles with a 3 bp deletion were found in the four individuals in which cDNA was analyzed, demonstrating that the 3 bp deletion alleles are transcribed and hence likely to be expressed. In humans non-classical MHC genes, (e.g. HLA-E, HLA-F, HLA-G and CD1) are expressed though they hold a lower diversity than classical MHC genes (HLA-A, HLA-B and HLA-C) [60]. MHC class I alleles containing a 3 bp deletion in exon 3 have been frequently reported among passerine birds, though not associated with the distinct characteristics described here [8, 61]. These previously reported 3 bp deletions within exon 3 are either placed at amino acid site 149 or 150 [34, 36]. However, in the sedge warbler, the 3 bp deletion is at position 126. This 3 bp deletion has so far not been reported in any other bird within the superfamily Sylvioidea or in any other songbird to date. Neither the 6 bp deletion nor the 3 bp deletion in the sedge warbler are within sites of the putative peptide binding region. The retained deletions, which preserve the open reading frame are likely to be at positions that do not alter the antigen binding ability of the MHC molecule. *In-silico* modelling of house sparrow and the tree sparrow MHC has demonstrated that the MHC molecules encoded by alleles bearing a 6 bp deletion display different antigen binding properties from those without such deletion [62]. Thus, it is possible that the MHC alleles containing deletions within exon 3 in sedge warblers also have altered peptide binding properties. Future studies involving *in-silico* modelling of sedge warbler MHC proteins would help to address this question.

## Conclusions

This study adds to the accumulating body of evidence suggesting that the MHC class I genes in passerine birds often are highly duplicated and highly polymorphic [7, 8, 24, 36, 38, 52, but see 63]. Although many passerine species appear to have highly duplicated MHC class I genes (notable examples include great tits and willow warblers with 19 MHC class I loci [8, 36]) the present study has extended the upper known limit to 33 MHC class I loci in a passerine bird. It has been suggested that having a high number of MHC genes could enable some of these genes to evolve different functions [64], and the extremely high number of MHC alleles in sedge warblers is consistent with the possibility that some MHC class I genes in this species may have derived a new function.

## Additional files

Additional file 1: Figure S1. Schematic overview of the primer design approach. PaDo primers, marked with red arrows, used for amplification and cloning of cDNA from 4 sedge warblers *Acrocephalus schoenobaenus* individuals. Hn primers (Westerdahl et al. 2004), designed for great reed warblers *A. arundinaceus*, used for obtaining whole exon 3 sequences in 4 individuals for both gDNA and cDNA. Subsequently sequences obtained for cDNA were used for designing specific sedge warbler primers (HnallaN and Hn46N) that amplify whole range of exon 3 MHC class I in sedge warblers, excluding a range of pseudogenes. (PDF 1364 kb)

Additional file 2: Table S1. Primers used to amplify MHC class I in sedge warbler. (DOCX 16 kb)

Additional file 3: Figure S2. Alignment of sedge warbler MHC class I amino acid sequences, covering the α_1_, α_2_ and α_3_ regions (species-specific nomenclature and GenBank accession numbers are used, Acsc-UA), in comparison with great reed warblers (Acar cN3, AJ 005503), white-throated sparrows *Zonotrichia albicollis* (XM005497294), Atlantic canaries *Serinus canaria* (XM009100025), golden-collared Manakins *Manacus vitellinus* (XM008930636), medium ground-finches *Geospiza fortis* (xm014311325), zebra finches *Taeniopygia guttata* (XM002186531), hooded crows *Corvus cornix cornix* (XM010392747), numbered according to full-length chicken MHC class I. Identity with sequence Acar cN3 is indicated with dots, codons corresponding to the PBR with (P). (PDF 189 kb)

Additional file 4: Figure S3. The distributions of the number of positively selected sites detected using the MEME test performed on subsets of 100 alleles randomly subsampled from alleles with no deletions (a) and alleles containing a 6 bp deletion (b). (DOCX 273 kb)
