# Supplementary material for: Extreme MHC class I diversity in the sedge warbler (Acrocephalus schoenobaenus); selection patterns and allelic divergence suggest that different genes have different functions
Source: BMC Evol Biol. 2017 Jul 5;17:159. doi: 10.1186/s12862-017-0997-9 (PMC5497381; doi:10.1186/s12862-017-0997-9)

Figure S1. Schematic overview of primer design approach. PaDo primers, marked with red arrows, used for amplification and cloning of cDNA from 4 sedge warbler *Acrocephalus schoenobaenus* individuals. Hn primers (Westerdahl *et al.* 2004), designed for great reed warbler *A. arundinaceus*, used for obtaining whole exon 3 sequences in 4 individuals for both gDNA and cDNA. Subsequently sequences obtained for cDNA were used for designing specific sedge warbler primers (HnallaN and Hn46N) that amplify whole range of exon 3 MHC class I in sedge warbler, excluding a range of pseudogenes.

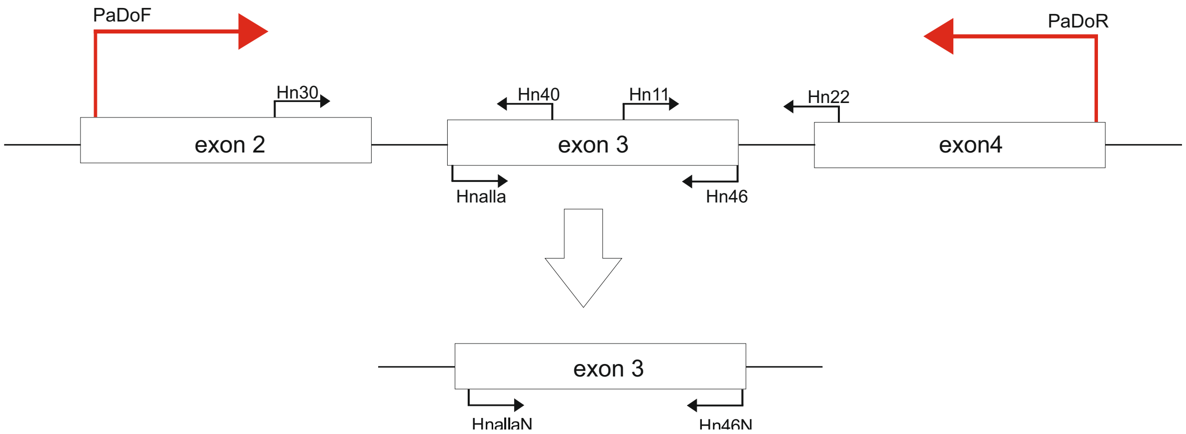

Supplement: Supplementary file 1 — Schematic overview of the primer design approach. PaDo primers, marked with red arrows, used for amplification and cloning of cDNA from 4 sedge warblers Acrocephalus schoenobaenus individuals. Hn primers (Westerdahl et al. 2004), designed for great reed warblers A. arundinaceus, used for obtaining whole exon 3 sequences in 4 individuals for both gDNA and cDNA. Subsequently sequences obtained for cDNA were used for designing specific sedge warbler primers (HnallaN and Hn46N) that amplify whole range of exon 3 MHC class I in sedge warblers, excluding a range of pseudogenes. (PDF 1364 kb) [file 12862_2017_997_MOESM1_ESM.pdf]
