# Supplementary material for: Extreme MHC class I diversity in the sedge warbler (Acrocephalus schoenobaenus); selection patterns and allelic divergence suggest that different genes have different functions
Source: BMC Evol Biol. 2017 Jul 5;17:159. doi: 10.1186/s12862-017-0997-9 (PMC5497381; doi:10.1186/s12862-017-0997-9)
Supplement: Supplementary file 2 — Primers used to amplify MHC class I in sedge warbler. (DOCX 16 kb) [file 12862_2017_997_MOESM2_ESM.docx]

Table S1. Primers used to amplify MHC class I in sedge warbler.

| Primer name | Primer sequence 5’-3’ | Reference |
| --- | --- | --- |
| HN11 | AGCGCTGCTGAGATCACCA | Westerdahl *et al.* 2004 |
| HN22 | CAAGATCAGCGTCCCGTGTT | Westerdahl *et al.* 2004 |
| HN30 | GAATATTGGGATAGGAAC | Westerdahl *et al.* 2004 |
| HN40 | CAGGTAATTCGTCCAATG | Westerdahl *et al.* 2004 |
| HN46 | ATCCCAAATTCCCACCCACCTT | Westerdahl *et al.* 2004 |
| HNalla | TCCCCACAGGTCTCCACAC | Westerdahl *et al.* 2004 |
| HNallaN | GAGYGGGGGTCTCCACAC | This study |
| HN46N | TGCGMTCCAGYTCCTTCTGCCC | This study |
