# Supplementary material for: Extreme MHC class I diversity in the sedge warbler (Acrocephalus schoenobaenus); selection patterns and allelic divergence suggest that different genes have different functions
Source: BMC Evol Biol. 2017 Jul 5;17:159. doi: 10.1186/s12862-017-0997-9 (PMC5497381; doi:10.1186/s12862-017-0997-9)
Supplement: Supplementary file 3 — Alignment of sedge warbler MHC class I amino acid sequences, covering the α1, α2 and α3 regions (species-specific nomenclature and GenBank accession numbers are used, Acsc-UA), in comparison with great reed warblers (Acar cN3, AJ 005503), white-throated sparrows Zonotrichia albicollis (XM005497294), Atlantic canaries Serinus canaria (XM009100025), golden-collared Manakins Manacus vitellinus (XM008930636), medium ground-finches Geospiza fortis (xm014311325), zebra finches Taeniopygia guttata (XM002186531), hooded crows Corvus cornix cornix (XM010392747), numbered according to full-length chicken MHC class I. Identity with sequence Acar cN3 is indicated with dots, codons corresponding to the PBR with (P). (PDF 189 kb) [file 12862_2017_997_MOESM3_ESM.pdf]

Figure S2. Alignment of sedge warbler MHC-I amino acid sequences, covering the $\alpha_1$, $\alpha_2$ and $\alpha_3$ regions (species-specific nomenclature and GenBank accession numbers are used, Acsc-UA), in comparison with great reed warbler (Acar cN3, AJ 005503 ), white-throated sparrow *Zonotrichia albicollis* (XM005497294), Atlantic canary *Serinus canaria* (XM009100025), golden-collared Manakin *Manacus vitellinus* (XM008930636), medium ground-finch *Geospiza fortis* (xm014311325), zebra finch *Taeniopygia guttata* (XM002186531), hooded crow *Corvus cornix cornix* (XM010392747), numbered according to full-length chicken MHC-I. Identity with sequence Acar cN3 is indicated with dots, codons corresponding to the PBR with (P).

```
                       α1                                                                                            α2
                              P P P            P P P                               P   P  PP  P  PP     PP  P         P P P
                                  10        20        30        40        50        60        70        80        90       100
                          ....|....|....|....|....|....|....|....|....|....|....|....|....|....|....|....|....|....|....|....|
Acrocephalus arundinaceus VLHSLHYLDVAVSEPSPGIPQFVAMGFVDGIPFTRYDSERGRMEPLTEWIKDSADPEYWDSQTQIGVGSQHVYARSLETLRERYNQSGGLHTVLRVYGCE
KM014676_Acsc-UA*01       ---------.......L...K.ME.................QV....Q.M..G.E.....RE..LL..R...N..A.QI.............W.......
KM014677_Acsc-UA*02       ---------............Y...........V........V....Q.V..........RN...S.RN......D....................V...
KM014678_Acsc-UA*03       ---------.G..D..S....YME..................E....P........G...RN..RA.QN...F..N..................N.....
KM014679_Acsc-UA*04       ---------.G...........M.I........V....D..Q.....Q.M..G.E..........C.RN...T..D...V................S...
KM014680_Acsc-UA*05       ---------..............S......T................Q.M..G.E.G...W...RC..W.VLD..N....Q.............QS....
KM014681_Acsc-UA*06       ---------.............LS.......................Q.M......G...W....N.QN...N..D...VP.............K.F...
KM014682_Acsc-UA*07       ---------............YMS.........V.F...........Q....GVE.....T....L.R....T..N...VL...........R.QS....
KM014683_Acsc-UA*08       ---------.............ME.........V.............Q....G.E.G...W....S..N..W..SN...AQ......R....W...I..D
KM014684_Acsc-UA*09       ---------.V.....L.....MS..................V....Q.M..G.E.G...RN...N.QN...F..N..IA............W...S...
KM014685_Acsc-UA*10       ---------.......L....YMVL.I......V.....W..A....Q.M..G.E.G.......RALWH...T..EQD..PDW.........W...S...
KM014686_Acsc-UA*11       ---------..............SI........V........K....Q.M..G.E.G...RS..NNERK.Q.F..N...A...........A...S....
KM014687_Acsc-UA*12       ---------.......L....YMVL.I......V........V....Q.M..G.E.G.......TA.QN..MF..N.K.VP...........W...I...
Zonotrichia albicollis    ..........G..........YMSL.YL.....A........V....Q....G.E....EG....N.EN...A..N..I.............R.W.....
Serinus canaria           ........T.........V..YMSI.................V....Q....G.E.G..EE....NERN..IN.GN.................QWL...D
Manacus vitellinus        ....MR..E...T.........MVL.Y......E........V....P.MAAG.E.....RE...DK.N.L.A.YK.DVG.D...W......WQ..A..D
Geospiza fortis           L....R..Y...P...L.....M.I.YL.....V.............Q.M..GVELG...MG...N.RN..ID..N...A............S.T....D
Taeniopygia guttata       -.......H.........V...TSI........V........A....Q.M..G.E.G...EE...S.RN...D..N....Q......R....A.WA...D
Corvus cornix cornix      .....R..S......G......TS...L.....Q........D....P.MEEGPK.G....E.ETSKR..QMD.VN...A.G..........LQ.DC..D

                                                                                                             α3
                                     P P                          P  PP     P  PP  P   P   P   P
                                 110       120       130       140       150       160       170       180       190       200
                          ....|....|....|....|....|....|....|....|....|....|....|....|....|....|....|....|....|....|....|....|
Acrocephalus arundinaceus LLSDGSVRGSERFGYDGRDFISF-DLESGRFMAADSAAEITRRRWEHEGIVAERQTNYLKHECPEWLQKYVGYGQKELERKEPPDVHVSGKEEHGTLILS
KM014676_Acsc-UA*01       ...........QH..N.......-.......V.............K...T....L..........E.......................R..........
KM014677_Acsc-UA*02       ......I...F.D..........-..G....V.................AE...W......I.......H.K............................
KM014678_Acsc-UA*03       .........TD.V..........-E.G.R..VP..........L..--.TE.DYL......D..D...................................
KM014679_Acsc-UA*04       ............H..........-..G....V...............D..E...L.............................................
KM014680_Acsc-UA*05       .....N....Y.D..........-E.G....VP...G........D....E...RI........D...................................
KM014681_Acsc-UA*06       ..........Y.D....Q.....-....R..V.................AE...L......I..K...................................
KM014682_Acsc-UA*07       ......I..TD.V....Q.....-.......V.................T....F......V......................................
KM014683_Acsc-UA*08       .....N.H.TF.N...R......-..G....V.................TM...WM.....V..Q...................................
KM014684_Acsc-UA*09       .......H.TY.Y..........-..G....V...........L...D.TE..GR......V......................................
KM014685_Acsc-UA*10       I...R.IH..F.V..N.......-....R..V.......S..........W...L......D.L.E..................................
KM014686_Acsc-UA*11       .........TS.N..N.......-.......V....T.........--..W..YL.........K........................R..........
KM014687_Acsc-UA*12       I.....I...Y......Q.....-....R..VP...G.................RM............................................
Zonotrichia albicollis    ..........Y.D..........-.......VP...G............-E...R......I.........R..................R......T..
Serinus canaria           ..........T.E....Q.L...-..GF...V....VG..S.....Q..-...HW......V.........................M..R.........
Manacus vitellinus        .........FY.Y..........-E.G..S.V...G..Q..K.K...D........H...NI.....R......REA..................I.T..
Geospiza fortis           .......H..HQI......H..WT..GF...VL.T........L..QDLNGV.KR......V......RH.R.....A............R.........
Taeniopygia guttata       ..........R.Y..........-..G..K.L..............Q.D-M...LK.....K.....R.........................Y......
Corvus cornix cornix      .....R....Y.L....Q..L..-EPR.QS.V..N...QV.T.S.NSD.VTV.HW....E.I.T.G.....E...EA................F......
```

```
                                     210       220       230       240       250       260       270
                              ....|....|....|....|....|....|....|....|....|....|....|....|....|....|..
Acrocephalus arundinaceus     CHAYGFYPKTIAVNWMKGDEIWDQETEWGGVVPNSDGTFHTWARIEALPEEREQYRCRVEHPGMPEPGIFAW
KM014676_Acsc-UA*01           ........N..............................................-----------------
KM014677_Acsc-UA*02           .......................................................-----------------
KM014678_Acsc-UA*03           .R.....................................................-----------------
KM014679_Acsc-UA*04           ............I..........................................-----------------
KM014680_Acsc-UA*05           ...................................V...................-----------------
KM014681_Acsc-UA*06           ..V....................................................-----------------
KM014682_Acsc-UA*07           ...................................................Q...-----------------
KM014683_Acsc-UA*08           ........N..............................................-----------------
KM014684_Acsc-UA*09           ...................K...................................-----------------
KM014685_Acsc-UA*10           .......................................................-----------------
KM014686_Acsc-UA*11           ..V....................................................-----------------
KM014687_Acsc-UA*12           ............I..........................................-----------------
Zonotrichia albicollis        ........N....S....G.TL..........................................L.......
Serinus canaria               ........N..T.S....GDTL.....R..I....................................R....
Manacus vitellinus            ........GM.GI..L....LR........I.........S........G...........A..........
Geospiza fortis               ........N....S....G.TL........I.................L.....................T.
Taeniopygia guttata           ........N..T.S...ENATL...M....I....................W.....K.D....L...L...
Corvus cornix cornix          ..V.....RP...S.......R........I................R...W.........S........T.
```
