# Supplementary material for: Extreme MHC class I diversity in the sedge warbler (Acrocephalus schoenobaenus); selection patterns and allelic divergence suggest that different genes have different functions
Source: BMC Evol Biol. 2017 Jul 5;17:159. doi: 10.1186/s12862-017-0997-9 (PMC5497381; doi:10.1186/s12862-017-0997-9)

Figure S3. The distributions of positively selected sites detected by MEME test performed on 100 allele sets subsampled randomly form no deletion alleles (a) and 6bp deletion alleles (b).


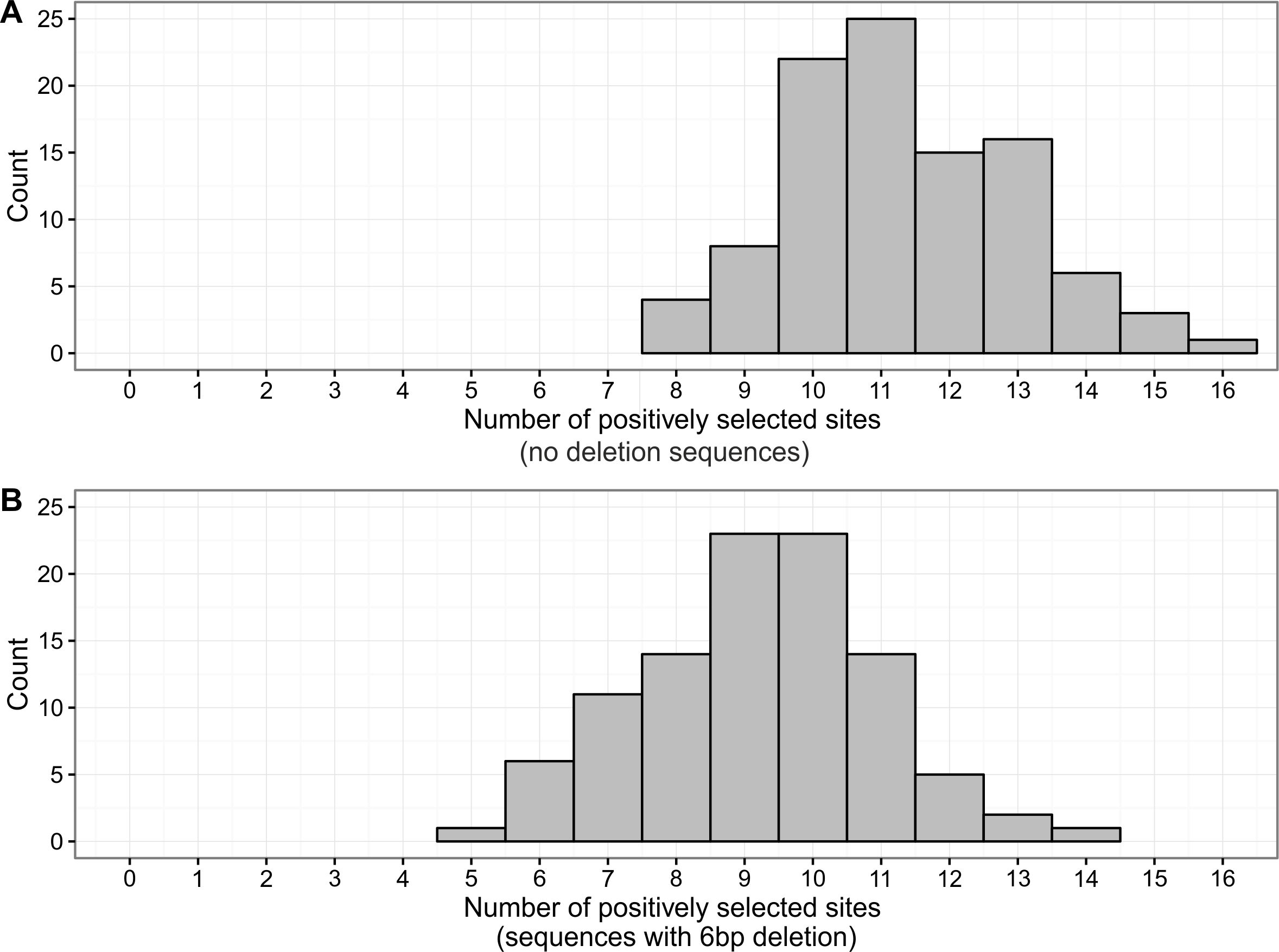

Supplement: Supplementary file 4 — The distributions of the number of positively selected sites detected using the MEME test performed on subsets of 100 alleles randomly subsampled from alleles with no deletions (a) and alleles containing a 6 bp deletion (b). (DOCX 273 kb) [file 12862_2017_997_MOESM4_ESM.docx]
